# The Distress Thermometer and Its Validity: A First Psychometric Study in Indonesian Women with Breast Cancer

**DOI:** 10.1371/journal.pone.0056353

**Published:** 2013-02-13

**Authors:** Aulia Iskandarsyah, Cora de Klerk, Dradjat R. Suardi, Monty P. Soemitro, Sawitri S. Sadarjoen, Jan Passchier

**Affiliations:** 1 Department of Psychiatry, Section Medical Psychology and Psychotherapy, Erasmus MC University Medical Center, Rotterdam, The Netherlands; 2 Department of Clinical Psychology, VU University, Amsterdam, The Netherlands; 3 Department of Clinical Psychology, Padjadjaran University, Bandung, Indonesia; 4 Department of Surgical Oncology, Hasan Sadikin Hospital, Bandung, Indonesia; The University of Hong Kong, Hong Kong

## Abstract

**Purpose:**

This study aims to translate the Distress Thermometer (DT) into Indonesian, test its validity in Indonesian women with breast cancer and determine norm scores of the Indonesian DT for clinically relevant distress.

**Methods:**

First, the original version of the DT was translated using a forward and backward translation procedure according to the guidelines. Next, a group of 120 breast cancer patients who were treated at the Outpatient Surgical Oncology Clinic in Hasan Sadikin Hospital in Indonesia completed a standard socio-demographic form, the DT and the Problem List, the Hospital Anxiety and Depression Scale (HADS) and the WHO Quality of Life (WHOQOL-BREF).

**Results:**

Receiver operating characteristic (ROC) curve analyses identified an area under the curve = 0.81 when compared to the HADS cutoff score of 15. A cutoff score of 5 on the DT had the best sensitivity (0.81) and specificity (0.64). Patients who scored above this cutoff reported more problems in the practical, family, emotional, spiritual/religious and physical domains (30 out of 36 problems, p-value<0.05) than patients below the cutoff score. Patients at advanced stages of cancer experienced more emotional and physical problems. Patient's distress level was negatively correlated with overall quality of life, general health and all quality of life domains.

**Conclusions:**

The DT was found to be a valid tool for screening distress in Indonesian breast cancer patients. We recommend using a cutoff score of 5 in this population.

## Introduction

In Indonesia, cancer incidence has an estimated number about 300,000 cases per year [1]. However, only 10% of these cases are treated in the health care system as the majority of these people do not seek medical help due to several factors, such as strong beliefs in traditional healers, fear and denial, and cultural taboos [2]–[3]. As one of the ten identifiable main diseases causing death in Indonesia [4], the diagnosis of cancer and its treatment often causes considerable psychological distress in patients. It has been recognized and reported in previous studies that 20–40% of cancer patients experience a significant level of distress [5]–[6]. Breast cancer is the primary cancer in Indonesia and its incidence and mortality rate is increasing [7]. Previous findings have shown that women with breast cancer experience psychological distress [8], even years after disease diagnosis and treatment [9]. Patients' distress is associated with a number of negative outcomes, including low adherence to treatment recommendation [10], poor satisfaction with care [11] and poor quality of life [12].

Similar to developed countries [13]–[14], distress among cancer patients often goes unrecognized by health care professionals in Indonesia. The ratio between the amount of health care professionals and cancer patients is still far from ideal in Indonesia. Data from 506 Government Hospitals in Indonesia showed that in average there are only 14 General Practitioners and 16 Specialists per hospital [4]. This condition may lead to several practical issues, including limited consultation time. In addition, a paternalistic style of doctor-patient communication and patients' unassertiveness are quite common in Indonesia [15]. These factors may also cause consultations to be focused primarily on physical aspects of cancer.

The National Comprehensive Cancer Network (NCCN) states that distress should be recognized, monitored, documented and treated promptly at all stages of the disease and in all settings [16]. Considering the high patients load and the unbalanced ratio between patients and health care professionals in Indonesia, there is an urgent need for a short and effective screening tool to detect distress among patients. Ideally, such a tool should be able to assess distress across the physical, psychological, social and spiritual domains [17]. As current screening tools are long and burdensome for patients to complete, there is a need for a brief, valid and easy to complete measure of distress in this population.

In order to meet this demand, the NCCN has developed the Distress Thermometer (DT) which is a single item that asks the patients to rate their distress using a visual analogue scale. It is accompanied by the Problem List (PL) that asks patients to identify any of 36 issues that have been a problem for them in the past week. The DT is very brief, easy to administer and it uses a word for psychological problems with non stigmatizing connotations, namely distress [16]. This tool was initially developed by the NCCN and many studies have reported that the DT is an effective screening tool for detecting distress among various medical conditions, such as prostate carcinoma [18], bone marrow transplantation [19], lung cancer [20], breast cancer [8] and mixed site cancer [21]. The NCCN suggests that a score of 4 or higher on the DT indicates a clinically significant distress level [16]. Some validation studies using the Hospital Anxiety and Depression Scale (HADS) found the same cutoff score of 4 [22]–[24], whereas other authors found that a cut off score of 5 [25]–[28] best distinguished distressed patients from non-distressed ones. Most studies found that DT scores above the cutoff are correlated with emotional, family and physical problems as measured by the Problem List. However, results on spiritual and religious concerns are inconclusive [21]–[23].

The Distress Thermometer has been successfully translated from English into several languages, such as Arabic [29], Dutch [30], Japanese [25], Korean [23], Turkish [24] and Italian, Spanish and Portuguese [27], but it has not yet been used in Indonesian cancer patients. Therefore, this study aims to translate the DT into Indonesian, test its validity in Indonesian women with breast cancer by comparing it with a well-established distress measure, i.e. the HADS, and to determine norm scores of the Indonesian DT for clinically relevant distress. The other aim was to establish the validity of the DT by examined its associations with the Problem List scores, socio-demographic and clinical characteristics, and quality of life.

## Methods

### Participants

Consecutive sampling was used to recruit 120 women with breast cancer from the outpatient surgical oncology clinic at Hasan Sadikin Hospital (HSH) Bandung in two phases. The first group of 50 patients was recruited between April–June 2010; the second group of 70 patients was recruited between June–October 2011, due to logistical reasons. Inclusion criteria were age ≥18 years, first diagnosis of breast cancer and adequate command of the Indonesian language. Patients who had been treated by psychiatrists were excluded from the study.

### Ethics statement

The study was approved by the Indonesian medical ethical committee and the Board of Directors of Hasan Sadikin Hospital. All samples were obtained with written informed consent reviewed by the ethical board.

### Procedures

This validation study was part of a larger investigation in which the correlates of non-adherence behavior in Indonesian breast cancer patients were explored. After receiving written permission from the NCCN, we used the forward and back translation method to translate the DT, since this method is the most frequently recommended and used method in translation guidelines for cross-cultural studies [31]. One of the authors of this study (A.I) who is a clinical psychologist translated the DT from English into the Indonesian language; the back translation into English was carried out by an English language teacher (J.H) who is a Native American who speaks the Indonesian language fluently and who has been living in Indonesia for 6 years. Upon completing the translation, a linguist (A.C) examined the original English version and the back translation version of the DT to assess the significance of any discrepancies. After some discussions with A.C, we finalized the Indonesian version of the Distress Thermometer.

A member of the administration staff of HSH identified eligible patients, explained the study purpose to them and asked for their initial consent to participate. One week later, those who wanted to participate were approached by one of the research assistants before their next visit to their physician. Ten master's students in clinical psychology were trained as research assistants and were supervised by S.S (clinical psychologist) and A.I. The research assistant provided further information about the study and instructions on how to fill in the questionnaires. After informed consent had been obtained, participants filled in the DT, the HADS, the World Health Organization Quality of Life (WHOQOL-BREF) and a demographic/background data form. Participants filled out the questionnaires in the waiting room before their consultations. Ten of the participants were illiterate, but they were able to speak and understand the Indonesian language. In these cases, the research assistants read both the informed consent form and the questionnaires out loud. After the participants signed the informed consent form, the research assistants helped them to fill in the questionnaires.

### Measures

#### Socio-demographic and medical status

A standard socio-demographic form was used to collect self-report data on age, marital status, education level, employment status, insurance status and family history of breast cancer. The patients' medical status, such as type and stage of cancer as defined by the TNM stadium classification system [32], type of treatment and time since diagnosis were obtained via a medical chart review.

#### Distress Thermometer (DT)

The DT is a 1-item, self-report measure of psychological distress developed by the NCCN [16]. Patients are asked to rate their distress in the past week on an 11-point visual analogue scale ranging from 0 (no distress) to 10 (extreme distress). Afterwards, patients are asked to fill in the Problem List (PL) that accompanies the visual image of the DT to check whether or not (yes/no) they experienced any of the problems listed during the previous 7 days. The PL version used in this study consisted of 36 problems that were grouped into five categories, namely practical problems, family problems, emotional problems, spiritual/religious concerns and physical problems. The PL aims to better define the nature of the problems which possibly cause the reported distress. To assess its association with the DT scores, the total amount of problems checked was calculated (range 0–36).

#### Hospital Anxiety and Depression Scale (HADS)

The HADS is a 14-item self-report questionnaire that has been developed to assess psychological distress in people with medical illness [33]. It consists of 2 subscales; one subscale consists of 7 items to measure anxiety (HADS-A) and one subscale consists of 7 items to measure depressive symptoms (HADS-D). Respondents are asked to indicate which of 4 options (rated 3-0) best describes their feelings during the previous week, which results in a maximum score of 21 on each subscale. The sum scores of the two subscales can be added up to a total score (HADS-T). The HADS has been widely used to validate the DT because of the similarity in their conceptual background [18], [22]–[27], [30], [34]–[35]. The HADS is available in the Indonesian language, but has not yet been psychometrically validated in Indonesian patients and cut-off scores for clinically relevant symptoms are not yet available. Therefore, in the present study we used the global cutoff score of the HADS total (≥15) that in studies elsewhere distinguished best between people with and without clinically significant emotional distress [36]–[37]. Factor analysis of the Indonesian version of the HADS demonstrated a two factor solution in good accordance with the HADS-A and HADS-D subscales, except for item 3: I feel cheerful and item 4: I feel as if I am slowed down. The solution accounted for 45% of variance. Both subscales were found to be internally consistent, with values of Cronbach's coefficient (alpha) being 0.77 and 0.74, respectively.

#### World Health Organization Quality of Life (WHOQOL-BREF)

The WHOQOL-BREF was developed as an abbreviation of the WHOQOL-100 to provide a short form quality of life assessment [38] It was developed by the WHO through a multicentre field trial situated within 23 countries. This tool is a self-report questionnaire which consists of 26 items, each item representing one facet of life that is considered to have a contribution to a person's quality of life. Twenty-four items measure four broad domains, namely physical health (e.g. mobility, pain and discomfort; 7 items), psychological health (e.g. body image and appearance, negative feelings, self esteem; 6 items), social relationships (e.g. personal relationships, social support; 3 items) and environment (e.g. financial resources, health and social care, physical environment; 8 items). Two other items measure the overall perception of quality of life and general health. The WHOQOL-BREF employs a 5-points scale (1 to 5) with a higher score indicating a higher level of self-perceived quality of life. The WHOQOL-BREF is available in a validated Indonesian version [39].

### Data Analysis

We used the Statistical Package for Social Science (SPSS 17.0) for data analysis. The mean score, the standard deviation, the median score and the frequency distribution of the DT were explored using descriptive statistical analysis. The concurrent and convergent validity of the DT with the HADS and the WHOQOL-BREF were examined by Pearson's correlation coefficient analyses. Receiver operating characteristic (ROC) analysis was used to identify the optimal DT cutoff score for distinguishing whether a patient experiences clinically significant distress as defined by the HADS. The Area Under the Curve (AUC) was used to estimate the overall discriminative accuracy of the DT cutoff score relative to the established cutoff score of the HADS≥15. We used a qualitative guideline for interpreting AUC values by Hosmer and Lemeshow [40], namely AUC = 0.50 as an indication that the test has no discrimination, AUC≤0.70 as an acceptable discrimination, AUC≤0.80 as a good discrimination and AUC≤0.90 as an excellent discrimination. ROC curves were used to show the trade-off between the sensitivity (true-positive rate) and specificity (true-negative rate) for every possible cutoff score of the DT.

To explore the association between the DT cutoff score and the Problem List, the demographic variables and the clinical variables, Chi-square analyses were conducted for categorical variables and *t*-test analyses were conducted for continuous variables. The association between the DT and the total score in the PL was explored by Pearson's correlation coefficient; associations between the DT cutoff scores and individual items in the PL were explored by the Chi-square analyses.

## Results

### Demographic and clinical characteristics

A total of 120 patients participated in this study. The response rate was 91%. Twelve out of 132 women approached declined to participate because they were too ill to fill in the questionnaires. As shown in Table 1, the mean age of the women in this sample was approximately 45.5 years of age (range; 28–66). Most of the participants were married (84%). The majority of the participants had middle school or lower education (i.e. 49% had elementary school, 20% had junior high school and 8% had no education). Seventy-three percent of the participants (73%) were housewives or unemployed. The mean number of months since diagnosis was 21.5 (*SD* = 20.3, range = 1–120 months). More than half of the study participants (52%) were in the disease stages III or IV. Fifty-six percent underwent mastectomy, 83% underwent chemotherapy and 23% underwent radiotherapy. Ninety-three percent of the participants had health insurance provided by the government to poor people (e.g. Jakesmas, ASKES, Gakin and Gakinda) and only 7% financed their own medical expenses. Twenty-five percent of the participants had a family history of breast cancer.

**Table 1:** Demographic and clinical characteristics of study participants.

Variable	n (%)
Age (*M±SD*)	45.5±8.04
Marital Status	
Married	101 (84%)
Single	2 (2%)
Divorced	0 (0%)
Widowed	17 (14%)
Education (highest)	
None	10 (8%)
Elementary school	59 (49%)
Junior high school	24 (20%)
Senior high school	21 (18%)
College or university	6 (5%)
Employment	
Housewife/unemployed	88 (73%)
Laborer/irregular job	25 (21%)
Private employee	2 (2%)
Government officer	5 (4%)
Months since diagnosis (*M±SD*)	21.5±20.3
Range (months)	1–120
Current stage of cancer	
1	3 (3%)
2	54 (45%)
3	46 (38%)
4	17 (14%)
Treatment	
Mastectomy	67 (56%)
Chemotherapy	99 (83%)
Radiotherapy	28 (23%)
Health insurance	
Yes	112 (93%)
No	8 (7%)
Family history of breast cancer	
Yes	30 (25%)
No	90 (75%)

### Average score on the DT and the Problem list

The average score of the patients on the DT was 4.7 (*SD* = 2.6). The most frequent problems checked in descending order in the practical domain were insurance/financial (60%), transportation (48%), housing (32%), work/school (24%) and child care (21%). The most frequently checked problems in the family problems category were: dealing with children (14%), the ability to have children (11%) and dealing with a partner (11%). In the emotional problems category, the most frequently checked problems were worry (81%), sadness (80%), fears (54%), depression (41%), nervousness (41%) and loss of interest in usual activities (33%). Eleven percent of the patients checked the item about spiritual/religious concerns. The ten most frequently checked problems in the physical problems category were pain (71%), fatigue (68%), nausea (55%), sleep (52%), getting around (51%), tingling in hands/feet (51%), eating (41%), appearance (36%), memory/concentration (36%) and skin dry/itchy (36%).

### Establishment of a DT cutoff score

**Figure 1:**
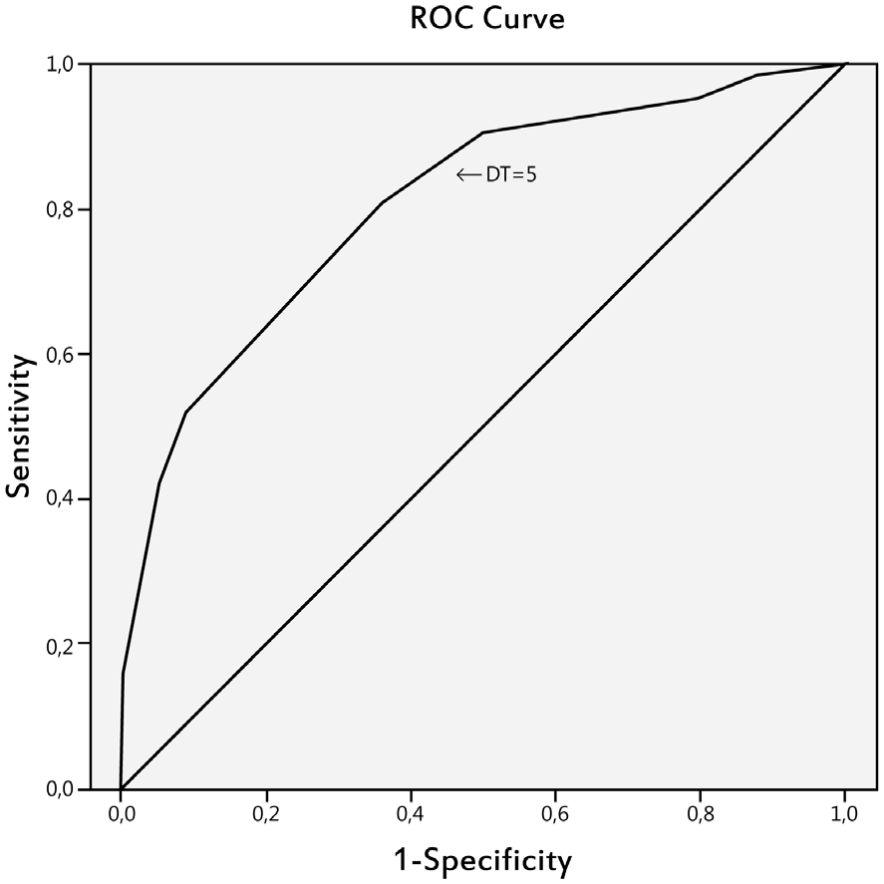
Receiving operation characteristic (ROC) curve of Distress Thermometer scores versus Hospital Anxiety and Depression Scale cutoff scores.

The Pearson's correlation coefficient between the DT scores and the HADS total was 0.58 (*p*<0.01); the correlation coefficients between the DT and the HADS-Anxiety and the HADS-Depression scales were 0.58 (*p*<0.01) and 0.48 (*p*<0.01), respectively. Using the HADS cutoff score of 15 as the criterion, sixty-two women (52%) were identified as experiencing clinically significant distress. The ROC analysis obtained the AUC of 0.81 (SE = 0.04; 95%CI = 0.73–0.88; *p*<0.001) (Figure 1). This AUC value indicates an excellent discrimination. Table 2 lists the Sensitivity, Specificity, Positive predictive values and Negative predictive value on each the DT cut-off point. A cutoff score of 5 on the DT optimally identified 81% of the HADS cases (sensitivity) and 64% of the HADS non cases (specificity) with positive and negative predictive values of 70% and 76%, respectively. Of those screened positive by the DT, 30% would be false positives and of those screened negative by the DT 24% would be false negatives.

**Table 2:** Sensitivity, Specificity, Positive and Negative predictive values on each the Distress Thermometer cut-off point.

Cut-off point	Sensitivity	Specificity	Positive predictive value	Negative predictive value
0/1	0.98	0.11	0.51	0.88
1/2	0.95	0.21	0.56	0.80
2/3	0.92	0.40	0.62	0.82
3/4	0.90	0.50	0.66	0.83
4/5	0.81	0.64	0.70	0.76
5/6	0.52	0.91	0.86	0.64
6/7	0.42	0.95	0.90	0.60
7/8	0.24	0.98	0.94	0.54
8/9	0.15	1.00	1.00	0.52
9/10	0.10	1.00	1.00	0.51

### Associations between the DT cutoff score and the Problem List items

The DT scores were statistically significantly correlated with the Problem List total score (*r* = 0.47, *p*<0.01). In the practical problems category (*p*≤0.05), the DT cutoff score was significantly associated with four problems (i.e. child care, housing, insurance/financial and work/school), and was not associated with transportation. The DT cutoff score was significantly associated with each of the problems in the family problems category (*p*≤0.05). Patients who scored above the cutoff experienced more problems in dealing with children, dealing with their partner and the ability to have children. In the emotional problems category (*p*≤0.05), the DT cutoff score was significantly associated with five problems (i.e. depression, nervousness, sadness, worry and loss of interest in usual activities), and was not associated with fears. Patients who scored above the cutoff experienced more spiritual/religious concerns (*p*≤0.05). Finally, in the physical problems category (*p*≤0.05), the DT cutoff score was significantly associated with 17 out of 21 problems (i.e. appearance, bathing/dressing, breathing, changes in urination, constipation, diarrhea, eating, fatigue, feeling swollen, fever, indigestion, memory/concentration, mouth sores, nose dry/congested, pain, sexual and skin dry/itchy), and was not associated with four other problems (i.e. getting around, nausea, sleep and tingling in hand/feet).

### Demographic and clinical characteristics associated with the DT cutoff score and the Problem List items

Marital status and insurance status were excluded in the Chi-square analyses, as some categories did not fulfill the minimum number of expected observations. We found that women with a score below the DT cutoff score of 5 did not differ significantly from women at or above the DT cutoff score of 5 on age, time since diagnosis, education, employment status and family history of cancer. However, we found a significantly difference in stage of cancer (χ^2^ = 3.90, df = 1, *p* = 0.048). Women with a score ≥5 were more likely to be at an advanced stage of cancer.

We found several significant associations between the PL-scores and the demographic and clinical characteristics. The advanced cancer patients (stage III or IV) had higher PL-total scores (*t* = −3.32, *p*<0.001), more emotional problems (*t* = −3.55, *p*<0.001) and more physical problems (*t* = −2.62, *p*<0.01) than the stage I or II cancer patients. Age was negatively correlated with physical problems (*r* = −0.21, *p*<0.05) and the PL-total scores (*r* = −0.182, *p*<0.05). PL scores were not associated with marital status, employment status, family history of cancer and time since diagnosis.

### The DT and the HADS correlations with the WHOQOL-BREF scores

Table 3 shows the correlation coefficients of distress and quality of life. The DT, the HADS total, the HADS Anxiety and the HADS Depression scores were significantly negatively correlated with overall quality of life, general health and all quality of life domains.

**Table 3:** Association between distress and quality of life.

	DT	HAD-A	HAD-D	HADS-T
Overall quality of life	−0.36**	−0.40**	−0.32**	−0.39**
General health	−0.43**	−0.44**	−0.31**	−0.41**
Physical health domain	−0.45**	−0.45**	−0.53**	−0.54**
Psychological domain	−0.55**	−0.55**	−0.53**	−0.59**
Social relationships domain	−0.22*	−0.29**	−0.38**	−0.35**
Environment domain	−0.31**	−0.30**	−0.36**	−0.36**

DT: Distress Thermometer; HAD-A: HAD Anxiety subscale score; HAD-D: HAD Depression subscale score; HADS-T: Hospital Anxiety and Depression Scale total score.

* Correlation is significant at the 0.05 level (2-tailed).

** Correlation is significant at the 0.01 level (2-tailed).

## Discussion

In this study, we examined the validity of the DT and its screening efficacy in detecting distress in Indonesian cancer patients. Our results showed that the Indonesian version of the DT has concurrent validity with the HADS, which is a well-established screening tool for distress. A cutoff score of 5 on the DT yielded optimal sensitivity and specificity. Patients who had a score above the cutoff score of 5 experienced more problems in the practical, family, emotional, spiritual/religious and physical domains than women with DT scores below this cut off score. Also, they were more likely to be at an advanced stage of cancer. Finally, distress as measured with the DT was found to be negatively correlated with overall quality of life, general health and all quality of life domains which establish the convergent validity of the Indonesian version of the DT.

The ROC analysis comparing the DT scores with the well-established HADS cutoff score of 15 obtained an AUC which indicates a good discrimination. Using the DT cutoff score of 5, eighty-one percent patients were identified correctly as being distressed and 64% identified correctly as not being distressed which is comparable to the result of the meta-analysis study by Mitchell [41]. This evidence shows that the DT has a screening efficacy for distress in Indonesian breast cancer patients. The current Distress Management Guidelines from the NCCN recommend that a DT score of 4 or higher indicates that a patient has a clinical significant level of distress and should be referred to a psychosocial care team [16]. However, we obtained a sensitivity of 90% and a specificity of 50% at a cutoff score of 4, resulting in a large proportion of patients incorrectly being identified as experiencing clinically significant distress. Considering the lack of health care professionals in Indonesia, we believe that it is more appropriate to use the cutoff score of 5 which yielded an optimal combination of sensitivity and specificity, to avoid a large number of false positive cases being diagnosed. Patients who may not require further intervention may feel burdened by further screening procedures. Moreover, false positive screening leads to higher health care costs and an increased need for health professionals. The DT cutoff score of 5 found in this study corresponds with the cutoff score found by other validation studies using the HADS [25]–[27], [34]–[35]

Patients who had significant distress were more likely to report more problems in the practical, family, emotional, spiritual/religious and physical domains. Interestingly, patients who had clinically significant distress were more likely to experience spiritual/religious concerns which is similar to the results of a study conducted in Korea [23]. In contrast, most studies conducted in Western countries found that clinically significant distress was not associated with spiritual/religious concerns [20], [22], [24], or only weakly related [34]. The significant correlation between high distress and spiritual/religious concerns is possibly due to the fact that Indonesian people are religious and have a strong belief in God. Many people rely on God to heal their disease. We hypothesize that people who do not feel any change in their illness will be more convinced their cancer as the will of God and they cannot change their destiny which in turn might trigger higher levels of distress.

Results of studies on associations between distress and socio-demographic and clinical characteristics in cancer patients are inconsistent [42]. In the present study, high distress was only found to be associated with stage of cancer, but not with other socio-demographic or clinical characteristics. This finding is concordance with previous studies that also unable to find significant associations between the DT and socio-demographic and clinical characteristics [18]–[19], [23]–[24], [27], [43]. Our finding that distress is associated with lower overall quality of life, general health and all quality of life domains is in line with the studies by Skarstein *et al.* [12] and Ozalp *et al.* [24], and further proves the validity of the Indonesian version of the DT.

The Problem List scores were associated with several demographic and clinical characteristics in the expected direction, suggesting that the Indonesian version of the Problem List is also a valid tool. Advanced cancer patients experienced more emotional problems and physical problems than patients at an early stage of cancer, and younger patients experienced more physical problems. These results are in line with previous studies results [44]–[45].

Several limitations of this study should be noted. Firstly, we used only breast cancer patients as our sample. Furthermore, we conducted this study at HSH which is a referral hospital that provides health services to the poor people. Therefore, the majority of the study participants had middle to low socio-economic and educational level. However, demographic and clinical characteristics of the patients (e.g. mean age, education level, marital status and stage of cancer) were similar to previous studies in Indonesian breast cancer patients [46]–[47]. Multi-center studies with a larger sample of various patient groups are needed to be able to extrapolate these results of the present study to other patient groups. Secondly, all measures used were self-rating questionnaires. Nevertheless, we included ten illiterate participants and they were helped to fill out the questionnaires. This may have led to some bias. Thirdly, the HADS Indonesian version has only been linguistically validated by the MAPI Institute which may have lead to some cultural bias. However, the basic psychometric examination results indicated that the HADS Indonesian version can be considered as a good instrument in terms of factor structure and internal consistency. Since the Geriatric Depression Scale, which is an instrument that is similar to the HADS has been shown to have the same optimal cut off point in both Western and Asian countries [48], we used the general HADS cutoff score suggested for Western countries in our study. Finally, this study examined the validity of the DT, but further research is required involving oncologists and nurses to confirm the feasibility of its use in daily care practice.

Bearing these limitations in mind, our findings suggest that the Indonesian version of DT could be used as a screening tool in daily cancer care in Indonesia. As the DT is brief and easy to administer, it might be an acceptable tool for oncologists in Indonesia. The NCCN suggests that early detection and treatment of distress leads to better adherence to treatment, better communication and prevents severe anxiety and depression [6]. According to our findings, cancer patients who experience distress above the DT cutoff score of 5 should be referred to a psychologist or another health professional to manage their distress and get appropriate treatment of their main distress sources as indicated in the PL. The use of the DT in daily cancer care in Indonesia may help oncologists to prevent potential severe psychological problems in cancer patients and provide additional interventions to patients who need it. Our results suggest that patients in an advanced stage of cancer should be given priority for psychological intervention. Such interventions are often part of medical psychology. Given that the field of medical psychology is new in Indonesia, we recommend its development by psychological faculties with academic hospitals in order to be able to provide adequate psychological resources to patients and doctors.

Our study did not only confirm the validity of the DT in Indonesian population, but also showed specific associations with several problems in the problem list. We found that women with breast cancer in Indonesia, most of whom are very religious, have different sources of distress than breast cancer patients in Western countries. In this respect, our study sheds light on cultural factors explaining cancer-related distress, thereby generating knowledge that is not only useful for physicians working in Asian countries, but also for physicians working with Asian populations in Western countries.
